# Transcriptional Landscaping Identifies a Beige Adipocyte Depot in the Newborn Mouse

**DOI:** 10.3390/cells10092368

**Published:** 2021-09-09

**Authors:** Anh Cuong Hoang, Haidong Yu, Tamás Röszer

**Affiliations:** Institute of Neurobiology, Ulm University, 89081 Ulm, Germany; anh.hoang@uni-ulm.de (A.C.H.); haidong.yu@uni-ulm.de (H.Y.)

**Keywords:** adipose tissue, adipogenesis, brown fat, thermogenesis, obesity

## Abstract

The present study sought to identify gene networks that are hallmarks of the developing inguinal subcutaneous adipose tissue (iWAT) and the interscapular brown adipose tissue (BAT) in the mouse. RNA profiling revealed that the iWAT of postnatal (P) day 6 mice expressed thermogenic and lipid catabolism transcripts, along with the abundance of transcripts associated with the beige adipogenesis program. This was an unexpected finding, as thermogenic BAT was believed to be the only site of nonshivering thermogenesis in the young mouse. However, the transcriptional landscape of BAT in P6 mice suggests that it is still undergoing differentiation and maturation, and that the iWAT temporally adopts thermogenic and lipolytic potential. Moreover, P6 iWAT and adult (P56) BAT were similar in their expression of immune gene networks, but P6 iWAT was unique in the abundant expression of antimicrobial proteins and virus entry factors, including a possible receptor for SARS-CoV-2. In summary, postnatal iWAT development is associated with a metabolic shift from thermogenesis and lipolysis towards fat storage. However, transcripts of beige-inducing signal pathways including β-adrenergic receptors and interleukin-4 signaling were underrepresented in young iWAT, suggesting that the signals for thermogenic fat differentiation may be different in early postnatal life and in adulthood.

## 1. Introduction

The field of adipose tissue biology is gaining ever more attention, largely driven by the alarming worldwide incidence of obesity and related diseases. Indeed, obesity is projected to affect ~60% of the global adult population by 2030 [1], and the prevalence of metabolic diseases associated with obesity––including insulin resistance, diabetes, metabolic syndrome and other immune-metabolic diseases––is also rising rapidly [2]. Beyond its traditional role as a lipid storage site, adipose tissue is pivotal in regulating the systemic metabolism [3]. Subcutaneous fat depots also serve as thermal insulation layers, and modulate endocrine and immune functions by producing and secreting various hormones, adipokines and cytokines [3]. These signals, in turn, affect the metabolic health of distant organs/tissues such as liver, muscle, endocrine pancreas, and the cardiovascular and skeletal systems [2]. Excess adipose tissue development leads to obesity, which can ultimately result in various chronic, incurable diseases such as coronary heart disease, non-alcoholic fatty liver disease, renal and retinal vascular complications, insulin resistance and diabetes [1,4,5]. Depending on its anatomical location, adipose tissue may serve additional functions; for example, it supports the subcutaneous connective layers and shapes body surfaces (e.g., Bichat fat pad at the cheek [6]), it helps to reduce mechanical stress on muscles and protect deep nerves, and it forms fat pads in the plantar and gluteal regions to support locomotion [7]. Adipose tissue also helps manual abilities and to firmly fix small objects by forming fat pads on the palmar surface of the hand [8].

Traditionally, two types of adipose tissue have been distinguished based on their cell morphology and functions: white adipose tissue (WAT) and brown adipose tissue (BAT) [9,10]. Adipocytes of the WAT are adapted for the long-term storage of fat and have the capacity to accumulate neutral lipids in a large droplet, which are broken down in response to hormonal signals under starvation [3]. By contrast, adipocytes of the BAT actively break down fat and oxidize fatty acids into ATP or heat [11,12,13]. BAT adipocytes are rich in mitochondria, and lipids are found in multiple small droplets surrounded by mitochondria [10,14,15,16]. BAT is also rich in glycogen, which, in addition to neutral lipids, serves as a metabolic fuel in cold-challenged BAT adipocytes [10,14,15,16]. Primordial fat cells in WAT and BAT depots contain glycogen and neutral lipids at birth [17,18,19], and these energy reserves comprise 1% and 16% of the body weight, respectively, in a newborn human [18]. Adipocyte lipolysis occurs rapidly after birth, followed by de novo fatty acid synthesis and lipogenesis that is accompanied by the catabolism of glycogen (so-called glycophagy) [20]. Glycophagy promotes lipid droplet formation, and lipid droplets are formed within glycogen clusters in developing BAT adipocytes [17]. The build-up of lipid stores continues during infancy, and is fueled by lipids consumed from breast milk or formula milk [21]. It is plausible that the scattered and small nature of lipid droplets in BAT increases their accessibility for mitochondrial β-oxidation [9,22]. Indeed, the difference in lipid droplet morphology is a distinguishing hallmark of WAT and BAT, and WAT adipocytes are often termed as unilocular (meaning “with one site” of fat storage) while BAT adipocytes are multilocular (meaning “many sites” of fat storage) [9,10,20,23]. WAT is present in all extant vertebrates, whereas BAT develops only in mammals, and is a less prevalent fat depot than WAT [24]. BAT is also typically located in the interscapular region [13], although thermogenic fat is present in the supraclavicular region, around large arteries and in the kidney capsule [25,26,27]. Obesity develops as a result of the expansion of WAT and decreases the amount of BAT [28,29,30]. Pediatric obesity is a current public health challenge, and it is thought that the decline in BAT during growth accelerates the development of obesity in children and adolescents [31].

Recent studies have described a third type of adipose tissue, the so-called brown-in-white adipose tissue (“brite”), also named “diffuse BAT” (reviewed in [11]). It contains both multilocular and unilocular fat cells, has a higher degree of fat oxidation than WAT, and can generate heat through uncoupled electron transport activity, resembling the function of the BAT [32]. The multilocular, thermogenic and fat-oxidizing adipocytes of the brown-in-white adipose tissue are often termed as beige adipocytes [27,33]. Cold exposure, Th2 cytokines and β-adrenergic stimuli, high-caloric diet, thiazolidinedione, and certain lipid species can evoke beige adipocyte traits in WAT [27,34,35,36,37,38]. Notably, thermogenic fat depots in the supraclavicular region, around the large arteries and in the kidney capsule, have been recently considered to be formed from beige adipocytes rather from BAT [39,40,41]. It is still debated whether beige adipocytes develop from BAT preadipocytes within WAT depots [42], as suggested by the term “brite”, or through unique differentiation programs of the adipocyte precursors to produce fat storing or fat oxidizing/thermogenic fat cells [43]. The resulting beige adipocytes are distributed among fat-storing white adipocytes [44]. Single-cell RNA sequencing has revealed considerable differences between the individual fat cells in terms of their thermogenic potential [45], and the distinct fat depots contain ontogenetically-heterogenous adipocyte populations [46,47]. The unique metabolic characteristics of beige adipocytes––including their ability to burn off lipids as heat––make them attractive candidates in the treatment of obesity, and the development of therapeutic beige adipocytes is an intensive area of research [32,34,35,36,48]. 

Newborn mammals have the ability to dissipate the energy in lipids as heat, which is necessary to maintain their body temperature [25,49]. Indeed, the clade of mammals and their evolutionary forerunners are termed “thermolipials”, which means “animals with warm fat” [50]. Certain fat depots of infant mammals were traditionally described as BAT [9,10,25,26]; however, as our understanding of adipose tissue development and function has grown, it is clear that newborn mammals and human infants have BAT only in some specific anatomical sites (e.g., in the interscapular region, buccal fat pad, around the large thoracic arteries), which disappears or regresses by adulthood [11,31,51]. The thermogenic potential of the subcutaneous adipose tissue has also been shown in infancy [20], which is sustained by breast milk-derived lipid signals, and is rapidly lost when breastfeeding is discontinued [38]. Of note, the loss of the thermogenic fat in the subcutaneous fat depots is accelerated in childhood obesity [52], and obesity reduces the potential of beige adipocyte differentiation [53]. As thermogenic fat also has lipolytic activity and provides free fatty acids for energy production by the liver and by the BAT, the presence of thermogenic cells in the subcutaneous fat depot contributes to the intense fat oxidation observed after birth in mammals [14,19,25,54]. The similarities between subcutaneous WAT in the newborn and BAT make it plausible that the infant subcutaneous fat depot is actually composed of beige fat cells. Beige adipocytes have been mostly studied under induction conditions in adults [27,33,55], and much less is known about beige adipocytes in infancy. Moreover, while there are abundant data on the transcriptional changes of the adult adipose tissue induced by cold stress or thermoneutral environment [32,45], including at the single-cell level [45], there is a paucity of similar studies in newborns. 

To question whether newborn WAT is similar to beige adipocytes and to BAT, the present study was designed to survey the transcriptional landscape in the developing fat depots of mice, and to define hallmark gene networks and hub genes. The data presented here help to define a new form of fat depot, which appears in early postnatal development, and is markedly distinct from BAT. Obesity in childhood is associated with a loss of thermogenic fat cells, and a similar loss has been observed in a mouse model of childhood obesity [38,56]. Our study shows that the BAT-independent subcutaneous fat depot is a relevant thermogenic/fat-oxidizing adipose tissue in early postnatal development in the mouse.

## 2. Materials and Methods

### 2.1. Animals and Cells

We used male C57BL/6 mice (Charles River Laboratories, Wilmington, MA, USA), housed under specific pathogen free conditions. Legislation of tissue collection from animals was approved by the regional governmental ethics and animal welfare committee in Tübingen, Germany (#1206; #1441; #1492; #o.189-20; #o.232-1,2,4,5). Primary mouse preadipocytes from inguinal WAT (iWAT) were isolated by collagenase digestion and cell separation and were subsequently cultured, as described [57,58]. To ensure the depletion of adipose tissue macrophages (ATMs) from the harvested preadipocytes, we used magnetic bead cell purification of the stromal vascular fraction with an antibody against the F4/80 antigen (Miltenyi Biotec, Bergisch Gladbach, Germany) [59]. Preadipocytes were maintained in cell culture medium supplemented with 20 μg/mL insulin. To induce white adipogenic differentiation of the preadipocytes of the stromal vascular fraction, we treated the cells with 50 μM IBMX, 1 μM dexamethasone, 1 μM rosiglitazone and 20 μg/mL insulin (all from Merck Sigma-Aldrich, St. Louis, MO, USA), as described [38]. The 3T3-L1 preadipocyte cell line was obtained from the ATCC (Merck Sigma-Aldrich) and maintained as described [60].

### 2.2. Histology and Image Analysis

Adipose tissue samples from iWAT and interscapular BAT were fixed with 4% paraformaldehyde and embedded in paraffin, as described (1). Sections were stained with hematoxylin and eosin, or with periodic acid Schiff (PAS)-reagent (Carl Roth, Karlsruhe, Germany). Immunohistochemistry was performed on paraffin-embedded tissue sections, using a mouse/human UCP1 antibody raised in rabbit (Thermo Fisher Scientific, Rockford, IL, USA) or an anti-mouse/human GABA-A receptor raised in rabbit (Alomone Labs, Jerusalem, Israel). Beige fat area was measured with our custom-developed image analysis software (BeAR, [38]). 

### 2.3. Flow Cytometry Analysis

Immune cells of iWAT and BAT were isolated by collagenase digestion and cell separation and were analyzed as described [57,58]. ATMs were labeled for CD11b and F4/80 antigen and mast cells were labeled for CD117 and FCεR1a. FACS analysis was performed on a BD LSR II cytometer [59]. Flow Repository identifier of raw FACS data: #FR-FCM-Z236.

### 2.4. mRNA Analysis and Next-Generation Sequencing

Extraction of total RNA was performed as described [57] using samples obtained from at least three animals (Figure S1). Quality of the isolated total cellular RNA was controlled with denaturing gel electrophoresis to label ribosomal RNAs. Quantitative (q) PCR assays were performed on the Quantabio platform (Beverly, MA, USA), using *Bactin*, *Gapdh* and *Ppia* as references [38]. Next-generation sequencing (NGS) analysis was performed on the BGISEQ-500 platform by BGI Genomics Inc. (Cambridge, MA, USA), generating ~26.2 million reads per sample. Sequencing reads were checked to remove low-quality, adaptor-polluted and unknown base reads before downstream analyses. After filtering, the clean reads were mapped to the reference genome using HISAT [61]. On average, 96.8% of reads were mapped, and the uniformity of the mapping result for each sample suggested that the samples were comparable. We mapped clean reads to reference transcripts using Bowtie2, then calculated the gene expression level for each sample with RSEM [62,63]. Sequencing data saturation analysis was used to measure whether the depth of sequencing data was sufficient for bioinformatics analysis. Based on their gene expression levels, we identified differentially-expressed genes (DEGs) between sample groups. We used the DEseq2 algorithm to detect the DEGs [64]. We performed Gene Ontology (GO) classification and functional enrichment for biological function, pathway and biological process. For annotation of transcripts, such as enrichment of predicted transcription factor sites and additional pathway analysis, we used EnrichR, Panther and Interferome-2.0 [65,66,67]. Interactome maps of the DEGs were generated with the STRING Functional Protein Associations Network [68]. To reflect the gene expression correlation between samples, we calculated the Pearson correlation coefficients for all gene expression levels between each two samples. All samples were submitted to hierarchical clustering by the expression level of all genes. Materials and data are available for secondary use upon request. Raw NGS data are deposited at GEO with the accession number #GSE133500. For secondary analysis, we used our previously published NGS dataset, with accession number #GSE125405.

### 2.5. Data Representation and Statistics

Gene expression data are represented as mean ± s.e.m. in bar graphs. To visualize gene transcription differences between samples, transcript levels are indicated relative to reference genes, or the relative abundance of the transcripts. We used Venn diagrams to display uniquely expressed genes of the different sample groups and to visualize the number of genes specifically expressed in a given sample group or expressed in multiple samples groups. Volcano or scatter plots were used to show the number of DEGs in the various sample groups. In the volcano plots the X-axis represents the log_2_ transformed fold change of the DEGs; the Y-axis represents the −log_10_ transformed significance. In the scatter plots, the X-axis represents value A (log_2_ transformed mean expression level), and the Y-axis represents value M (log_2_ transformed fold change). Red data points represent upregulated DEGs. Blue data points represent downregulated DEGs. Gray data points represent non-DEGs. The applied statistical tests are indicated in the respective figure legends.

## 3. Results

### 3.1. Gene Networks and Hub Genes of Fat Depots during Early Life and in Adulthood

**Figure 1:**
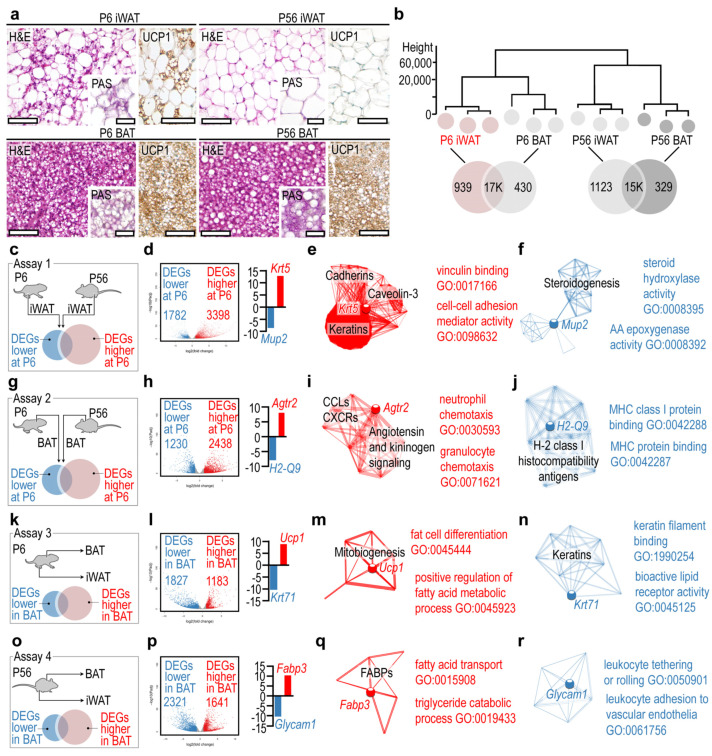
Gene networks and hub genes of the developing iWAT and BAT. (**a**) Hematoxylin and eosin (H&E) stained sections, and UCP1 immunostaining of BAT and iWAT at P6 and P56. Insets show periodic acid Schiff (PAS)-stained sections. Scale 50 μm. (**b**) Hierarchical clustering of samples. The closer the samples were to each other, the more similar their transcriptional landscapes were. (**c**) In assay 1, gene expression profiles of P6 and P56 iWAT were compared. (**d**) Volcano plot of DEGs. (**e**) Interactome map and most significant GO terms of DEGs overrepresented in P6 iWAT in assay 1. (**f**) Interactome map and most significant GO terms of underrepresented DEGs in P6 iWAT in assay 1. (**g**) In assay 2, gene expression profiles of P6 and P56 BAT were compared. (**h**) Volcano plot of DEGs. (**i**) Interactome map and most significant GO terms of DEGs overrepresented in P6 BAT in assay 1. (**j**) Interactome map and most significant GO terms of underrepresented DEGs in P6 BAT in assay 1. (**k**) In assay 3, gene expression profiles of P6 BAT and P6 iWAT were compared. (**l**) Volcano plot of DEGs. (**m**) Interactome map and most significant GO terms of DEGs overrepresented in P6 BAT in assay 3. (**n**) Interactome map and most significant GO terms of underrepresented DEGs in P6 BAT in assay 1. (**o**) In assay 4, gene expression profiles of P56 BAT and P56 iWAT were compared. (**p**) Volcano plot of DEGs. (**q**) Interactome map and most significant GO terms of DEGs overrepresented in P56 BAT in assay 4. (**r**) Interactome map and most significant GO terms of underrepresented DEGs in P56 BAT in assay 4.

We isolated adipose tissue depots from mice at postnatal day 6 (P6) and P56 for NGS analysis of the iWAT and the interscapular BAT. At P6, the iWAT was rich in multilocular fat cells and expressed uncoupling protein 1 (UCP1) (Figure 1a), the main hallmark of thermogenic fat. By contrast, P56 iWAT lacked multilocular fat cells and UCP1 expression (Figure 1a). Interscapular BAT was composed of multilocular cells at both P6 and P56 and showed strong UCP1 expression (Figure 1a, Figure S2). The cytoplasmic content of the fat cells in P6 iWAT, P6 BAT and P56 BATstained positive for PAS, indicating the presence of glycogen (Figure 1a), particularly in the multilocular adipocytes, in accordance with previous observations [17,69]. The extracellular matrix stained positive for PAS in all tested adipose tissue depots (Figure 1a). 

Hierarchical clustering analysis revealed that the transcriptional landscapes of iWAT and BAT were distinct at both P6 and P56 (Figure 1b). The least similarity was found between P6 iWAT and P56 BAT (Figure 1b), although both tissues contained multilocular adipocytes and expressed UCP1 (Figure 1a). The gene networks of each tissue were generated using GO terms and the Kyoto Encyclopedia of Genes and Genomes (KEGG) pathway assignment. The gene networks are accessible in an interactive format in Figure S3. P6 iWAT had less than 1K unique gene products, which were expressed specifically by this fat depot and not by the other depots analyzed (Figure 1b). We performed pairwise comparisons of P6 iWAT and P56 iWAT, and P6 BAT and P56 BAT to define DEGs that have a restricted expression in infancy and in adulthood (Figure 1c–f). When we compared P6 and P56 iWAT, we found that the former had >3.3K overrepresented DEGs and 1.7K underrepresented DEGs compared with P56 iWAT (Figure 1c). The most overrepresented DEG was *Krt5*, encoding keratin 5. Interactome analysis identified *Krt5* as a hub gene, interconnecting three extensive gene networks: a gene network of cadherins, a gene network of caveolin-3, and a gene network of keratins (Figure 1e, Table S1, *Krt5* network). Members of these networks were equally overrepresented in P6 iWAT. GO analysis indicated their involvement in vinculin binding and in cell−cell adhesion. The most underrepresented DEG in P6 iWAT was *Mup2*, encoding for major urinary protein 2, and it appeared to be a hub gene of a gene network of steroidogenesis (Figure 1f, Table S2, *Mup2* network). GO analysis indicated that the underrepresented DEGs of this network had a function in steroid hydrolase activity and in arachidonic acid metabolism. 

The pairwise comparison analysis of P6 BAT and P56 BAT (Figure 1d) revealed that 2.4K DEGs were overrepresented in P6 BAT, with *Agtr2*, encoding angiotensin II receptor type 2, having the highest expression level (Figure 1g,h). The networks associated with *Agtr2* included chemokine ligands (CCLs) and CXC chemokine receptors (CXCRs), and also elements of angiotensin- and kininogen-signaling (Figure 1i, Table S3, *Agtr2* network). Genes of these networks were also overrepresented in P6 BAT, and their GO terms were associated with neutrophil granulocyte chemotaxis. The most underrepresented DEG in P6 BAT was *H2-Q9*, which belongs to the H-2 and class I histocompatibility antigens network (Figure 1j, Table S4, *H2-Q9* network). Members of this network were underrepresented in P6 BAT and had GO terms associated with MHC class I protein binding.

The above two comparisons aimed to define hub genes and gene networks associated with the postnatal development of iWAT and BAT. We next sought to define hub genes and gene networks which were specific to the BAT, irrespective of postnatal age. We thus compared P6 BAT with P6 iWAT (Figure 1k) and P56 BAT with P56 iWAT (Figure 1f). We found that 1.1K DEGs were overrepresented in P6 BAT, with *Ucp1* showing the highest expression level (Figure 1l). Notably, *Ucp1* was not associated with extensive gene networks and was not defined as a hub gene; rather, it was a member of a small network of mitobiogenesis genes that were overrepresented in P6 BAT (Figure 1m, Table S5, *Ucp1* network), and with roles in fat cell differentiation and fatty acid catabolism (Figure 1m). A total of 1.8K DEGs were underrepresented at P6, with *Krt71* (keratin 71) showing the lowest expression level (Figure 1l). *Krt71* belongs to a small network of keratins, with GO terms associated with keratin filament binding and bioactive lipid receptor activity (Figure 1n, Table S6, *Krt71* network). Lastly, we compared P56 BAT with P56 iWAT (Figure 1o), which revealed 1.6K DEGs overrepresented and 2.3K DEGs underrepresented in P56 BAT (Figure 1p). The most overrepresented DEG was *Fabp3*, encoding fatty acid-binding protein 3, which was not positioned as a hub gene but belongs to a small network of fatty acid-binding proteins (FABPs) with functions in fatty acid transport and lipid catabolism (Figure 1q, Table S7, *Fabp3* network). The most underrepresented DEG was a pseudogene of the innate immune system, termed *Glycam1* (glycosylation-dependent cell adhesion molecule 1), belonging to a gene network of leukocyte tethering and adhesion (Figure 1r, Table S8, *Glycam1* network). 

### 3.2. Cell Cycle and Myogenesis Genes Are Hallmarks of the Developing BAT

**Figure 2:**
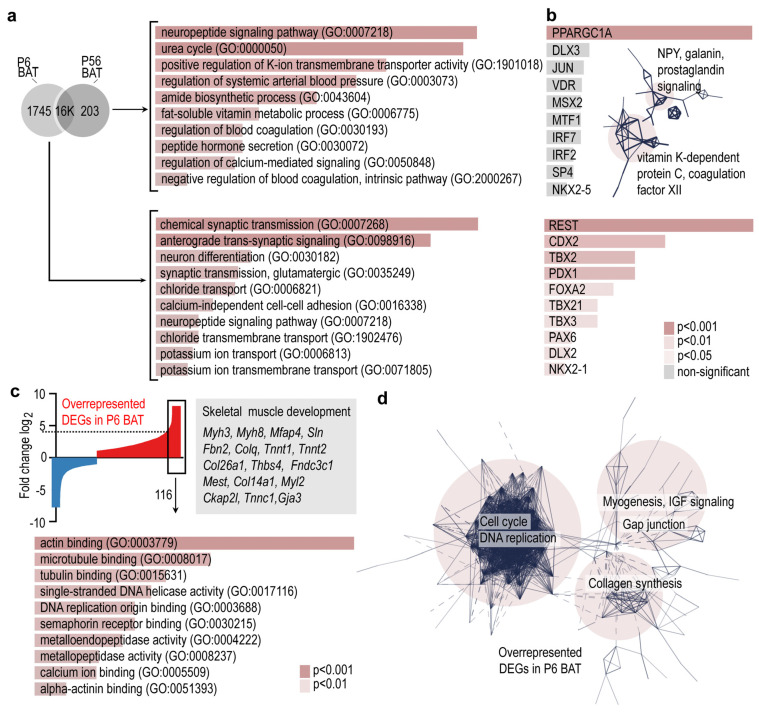
Gene ontology analysis and gene networks of BAT-specific transcripts. (**a**) Venn diagram showing uniquely expressed genes in P6 BAT or P56 BAT. GO terms associated with P6-, and P56-specific transcripts. (**b**) Transcription factor-binding prediction for P6-, and P56-specific transcripts. Inset shows gene networks of P56-specific transcripts. Combined *p*-values of the enrichment are shown in color codes in panels a and b. (**c**) Top: Gene expression profiles of P6 and P56 BAT were compared, and the differences were visualized on the Y-axis as log_2_ of fold change of DEGs. DEGs overrepresented at P6 are indicated in red, and underrepresented DEGs are shown in blue. The 116 DEGs which were ≥4.5-fold overrepresented in P6 were analyzed further. Bottom: GO terms of the 116 most overrepresented DEGs. (**d**) Interactome map of DEGs overrepresented in P6 BAT. PPI enrichment *p*-value < 1.0 × 10^−16^.

The analysis of gene networks suggested that there was a greater gene expression difference between P6 BAT and P56 BAT than between iWAT and BAT, pointing to a high degree of transcriptional changes associated with the postnatal development of BAT. Accordingly, we further analyzed the transcriptional landscape of BAT at P6 and P56. We identified only 0.2K genes that were unique to P56 BAT and 1.7K genes that were unique to P6 BAT (Figure 2a). Genes expressed only by the P56 BAT were associated with neuropeptide signaling, urea cycle, and metabolic processes of lipid-soluble vitamins and blood coagulation. Comprehensive analysis revealed that these genes were associated with neuropeptide Y (NPY), galanin and prostaglandin signaling, and with vitamin K-dependent protein C and coagulation factor XII (Hageman factor) (Figure 2b). Moreover, the genes characteristic to P56 BAT had an enriched predicted binding for peroxisome proliferator activator receptor gamma coactivator-1a (PPARGC1A) (Figure 2b). By contrast the P6-specific genes were associated with synaptic transmission, neuron differentiation, neuropeptide signaling and cell adhesion, and had predicted binding sites for various transcription factors, such as RE1-silencing transcription factor (REST), homeobox protein CDX-2 (CDX2) and T-box transcription factor 2 (TBX2) (Figure 2b), which may be associated with adipogenesis [70]. 

The most overrepresented DEGs of P6 BAT were associated with skeletal muscle development, such as myosin 3 *(Myh3)*, myosin 8 *(Myh8)*, microfibril-associated protein 4 *(Mfap4)*, sarcolipin *(Sln)*, fibrillin-2 *(Fbn2)* and many other genes of actin binding and microtubule binding (Figure 2c). These genes formed three interconnected networks, which were associated with cell cycle and DNA replication, myogenesis, insulin-like growth factor (IGF) signaling, collagen synthesis and gap junctions (Figure 2d). The transcriptional landscape of P6 BAT was associated with ongoing cell proliferation and tissue differentiation processes, with a notable overrepresentation of genes involved in skeletal muscle differentiation. While the demand of thermogenesis is higher in early postnatal life than in adulthood, we found similar levels of thermogenic genes and of genes associated with mitobiogenesis and fat catabolism between young and adult BAT. 

### 3.3. Neuropeptide and GABA Signaling Networks Are Similarly Overrepresented in Adult BAT and Young iWAT

**Figure 3:**
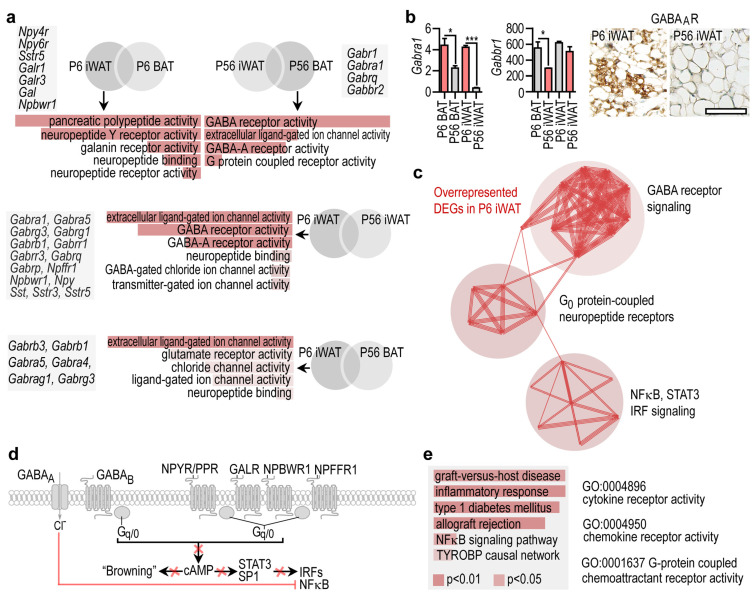
Gene expression pattern shared by P56 BAT and P6 iWAT. (**a**) GO analysis and representative gene transcripts specific to P6 iWAT and P56 BAT. (**b**) Left: Expression level of *Gabra1*, encoding GABA-A receptor, and *Gabbr1*, encoding GABA-B receptor in P6 iWAT, P6 BAT, P56 iWAT and P56 BAT. * *p* < 0.05, *** *p* < 0.001, Student’s two-tailed unpaired t-test. Right: Immunostaining of GABA-A in iWAT at P6 and P56, scale 50 μm. (**c**) Interactome map of DEGs overrepresented in P6 iWAT. PPI enrichment *p*-value < 1.0 × 10^−16^. (**d**) Scheme showing cell compartment association of DEGs overrepresented in P6 iWAT. cAMP is a known signal for transformation of WAT into beige adipocyte-rich depots, in the so-called adipose tissue “browning” process [35]. Red cross denotes inhibition of the respective pathways. (**e**) GO analysis of cellular functions and biological processes of immune-related transcripts in P56 BAT. Combined *p*-values are indicated with color codes. TYROBP: TYRO protein tyrosine kinase-binding protein.

Our results revealed that DEGs associated with neuropeptide signaling were overrepresented in P56 BAT. We next aimed to define these genes. Neuropeptide signaling-associated DEGs were overrepresented in P6 iWAT, making it similar to P56 BAT (Figure 3a). Specifically, the DEGs were associated with pancreatic polypeptide (PP) signaling, neuropeptide Y (NPY) signaling and gamma aminobutyric acid (GABA) receptor signaling. Further comparisons of P6 iWAT with P6 BAT, P56 iWAT with P56 BAT, P6 iWAT with P56 iWAT, and P6 iWAT with P56 BAT revealed that the genes of neuropeptide signaling and GABA receptor activity were overrepresented in both P56 BAT and P6 iWAT.

The DEGs included NPY and PP receptors (*Npy2r*, *Npy4r*, and the pseudogene *Npy6r*), neuropeptide B/W receptor *(Npbwr1)*, NPY *(Npy)*, galanin *(Gal)* and several components of GABA-A receptor signaling. GABA-A receptors were overrepresented in P56 BAT and P6 iWAT and minimally expressed in P56 iWAT, whereas GABA-B receptors were stably expressed at P6 and P56, and showed higher levels in iWAT than in BAT (Figure 3b, Figure S4). Interactome analysis of the DEGs overrepresented in P6 iWAT revealed their association with three interconnected networks: GABA receptor signaling, G_0_ protein-coupled neuropeptide signaling, and NFκB, STAT3, and interferon regulatory factor (IRF)-signaling (Figure 3c). Functional annotation of the DEGs indicated that they were all involved in inhibitory signaling towards NFκB, STAT3 and IRFs, suggesting a potential immune suppressor or anti-inflammatory role of these neuropeptides and GABA in P6 iWAT and P56 BAT (Figure 3d). In this line, inflammatory genes, interferon-regulated genes and the NFκB pathway were all underrepresented in P56 BAT (Figure 3e).

### 3.4. Transcriptional Landscapes of Immune Genes in the Developing Fat Depots

**Figure 4:**
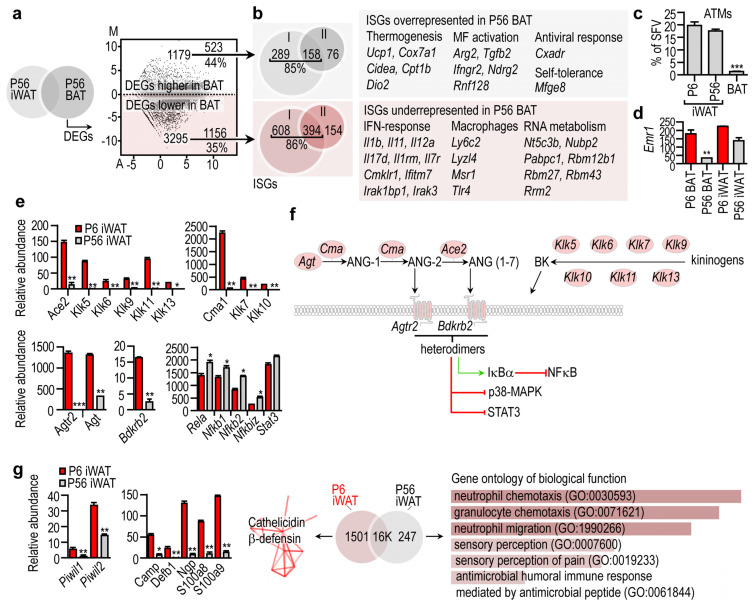
Immune-related gene transcripts in the BAT and in the iWAT. (**a**) P56 iWAT and P56 BAT were compared, and the DEGs were visualized in a scatter plot. Number of DEGs, and the percentage of interferon-stimulated genes (ISGs) are shown. (**b**) Representative ISGs overrepresented or underrepresented in BAT. (**c**) Percentage of adipose tissue macrophages (ATMs) within the stromal vascular fraction (SVF) of P6 iWAT, P56 iWAT and P56 BAT. *** *p* < 0.001, two-way ANOVA with Dunnett’s post hoc test, using P6 BAT as reference group (**d**) Expression level of the macrophage marker *Emr1*, encoding F4/80 antigen. ** *p* < 0.01, two-way ANOVA with Dunnett’s post hoc test, using P6 BAT as reference group. (**e**) Expression level of genes associated with the local angiotensin and kininogen system. * *p* < 0.05, ** *p* < 0.01, *** *p* < 0.001, Student’s two-tailed unpaired t-test. (**f**) Scheme summarizing the cell compartment association of the gene products associated with the local angiotensin and kininogen system. All indicated gene products were overrepresented in P6 iWAT. (**g**) Left: Expression level of PIWI protein-encoding genes, and genes encoding cathelicidin and β-defensins. * *p* < 0.05, ** *p* < 0.001, Student’s two-tailed unpaired t-test. Middle: Cathelicidin and β-defensin gene network overrepresented in P6 iWAT. PPI enrichment *p*-value < 1.0 × 10^−16^. Right: Gene ontology of DEGs overrepresented in P56 iWAT. Combined *p*-value < 0.01.

We further explored the expression of immune signaling genes in BAT and found that P56 BAT and P56 iWAT had distinct transcriptional profiles of interferon-stimulated genes (ISGs, Figure 4a, GO analysis and KEGG pathways are available in the Supplemental Material). Indeed, 44% of the DEGs overrepresented in P56 BAT were ISGs, including thermogenic genes such as *Ucp1*, mitobiogenesis genes such as *Cox7a1*, *Cidea*, *Cpt1b* and *Dio2,* encoding cytochrome c oxidase subunit 7A1, cell death inducing DFFA-like effector A, carnitine palmitoyltransferase 1B and iodothyronine deiodinase 2, respectively [71,72] (Figure 4b). Other ISGs that were overrepresented in BAT included M2 macrophage-activation genes such as arginase 2 *(Arg2)* and transforming growth factor beta *(Tgfb2)*, the M1 macrophage activation gene gamma interferon receptor *(Ifngr2)*, macrophage proliferation gene NDRG family member 2 *(Ndrg2)*, and the ubiquitin ligase ring-finger protein 128 *(Rnf128)* [57,73]. RNF128 is known to increase the ubiquitination and proteasomal degradation of phosphorylated STAT6, and hence can impede M2 activation of macrophages and Th2 signaling of lymphocytes [74]. Some virus entry factors and antiviral ISGs such as Coxsackievirus and adenovirus receptor *(Cxadr)*, and self-tolerance genes such as milk fat globule EGF and factor V/VIII domain containing protein *(Mfge8*), were overrepresented in P56 BAT (Figure 4b).

In turn, 35% of the underrepresented DEGs in P56 BAT were ISGs, including the IFN-response genes interleukin 1b *(Il1b)*, *Il11*, *Il12a*, interleukin-7-receptor *(Il7r)*, and macrophage genes such as lymphocyte antigen 6 *(Ly6c2)*, lysozyme like 4 *(Lyzl4)* and macrophage scavenger receptor 1 *(Msr1)*. The ISGs were type I and type II interferon target genes. RNA metabolism genes were also underrepresented in P56 BAT (Figure 4b). The evident lack of expressed macrophage genes in BAT was consistent with the paucity of adipose tissue macrophages (ATMs) (Figure 4c,d). 

When we extended our analysis to iWAT, we found that the *Agtr2* gene network was overrepresented in P6 iWAT, along with angiotensin converting enzyme 2 (*Ace2*) and bradykinin receptor 2 (*Bdkrb2*) (Figure 4e). ACE2 is a receptor for SARS-CoV-2 [75] and is believed to enhance virus entry into human adipocytes, which may account for the increased disease severity in patients with COVID-19 and obesity [76]. NGS analysis showed that *Ace2* was minimally expressed in iWAT at P56, but robustly expressed at P6. The *Agtr2* network included *Ace2*, angiotensin (*Ang*), chymase (*Cma1*) and genes encoding kallikreins (Figure 4f). As a gene network, these genes form a tissue hormone signaling niche and convert angiotensinogen into ANG-2 and ANG (1–7) [77] (Figure 4f). Heterodimers of AGTR2 and BDKRB2 phosphorylate IκBα and dephosphorylate STAT3 and p38-MAPK, and induce nitric oxide production in endothelial cells [78]. This signaling potentially inhibits NFκB and STAT3 signaling (Figure 4f). Coherently, iWAT maturation was associated with an increase of DEGs of neutrophil chemotaxis and the expression of inflammatory genes (Figure 4g). This indicates that the P56 iWAT environment is inflammatory, which is in line with previous observations showing that adipogenesis is associated with the upregulated expression of NFκB subunits [79] (Figure 4e). By contrast, P6 iWAT had a robust gene expression of the antimicrobial peptides cathelicidin and β-defensin (Figure 4g). Altogether, a local angiotensin and kininogen-kallikrein system along with the abundance of AGTR2 and BDKRB2 can be used as a hallmark of P6 iWAT, distinguishing it from both P56 iWAT and BAT.

### 3.5. Transcription of Beige Marker Genes in the Developing BAT and iWAT

**Figure 5:**
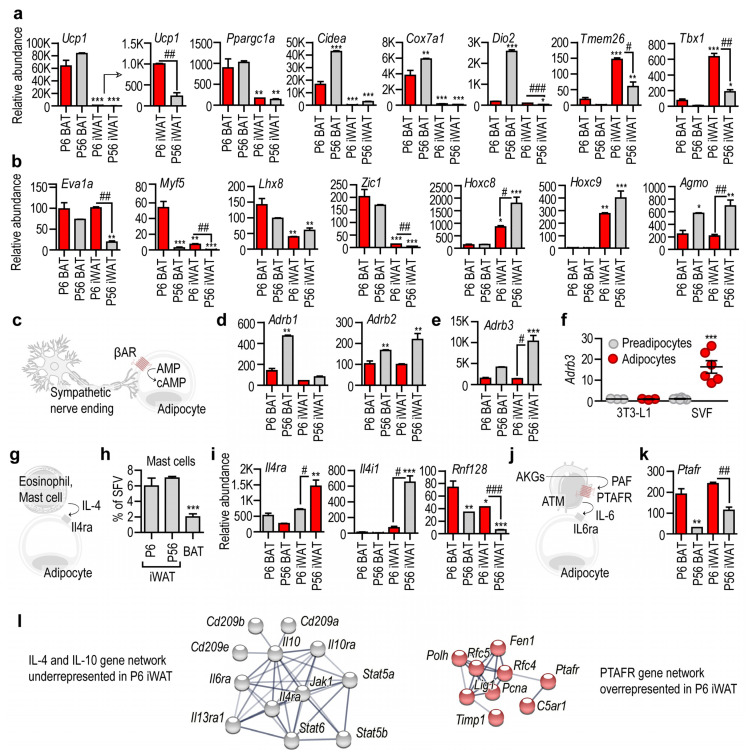
Transcription of beige-marker genes and beige-inducing signal pathways. (**a**,**b**) Transcript level of genes previously defined as markers of BAT and beige adipocytes. *Ucp1* is key for nonshivering thermogenesis; *Ppargc1a* is a regulator of mitobiogenesis; *Cidea*, *Cox7a1*, *Dio2*, *Tmem26* and *Tbx1* are markers of beige adipocytes; *Eva1a* is a marker of BAT; *Myf5* is expressed by progenitors of BAT adipocytes; *Lhx8* and *Zic1* are markers of BAT. The levels of *Hoxc8* and *Hoxc9* increase during WAT adipocyte development. *Agmo* is responsible for the degradation of beige adipocyte-inducing lipid species, so-called alkylglycerols (AKGs). (**c**) Scheme of beige induction by β-adrenergic stimuli of the sympathetic nervous system. (**d**) Level of β-adrenergic receptors. (**e**) Level of *Adrb3* in the various fat depots. (**f**) *Adrb3* level in 3T3-L1 preadipocytes and in in vitro cultured primary preadipocytes of the iWAT stromal vascular fraction (SVF). Adipocyte development was chemically induced. (**g**) Scheme of IL-4-induced adipose tissue browning. (**h**) Percentage of mast cells in the SVF of iWAT and BAT. (**i**) Expression level of IL-4 signaling in the various fat depots. (**j**) Scheme of adipose tissue browning induced in postnatal life by the AKG–PAF–IL-6 axis. (**k**) Expression of *Paftr*. (**l**) Underrepresented gene network of IL-4 signaling in P6 iWAT. Overrepresented gene network of PAF signaling in P6 iWAT. PPI enrichment *p*-value < 1.0 × 10^−16^. * *p* < 0.05, ** *p* < 0.01, *** *p* < 0.001, two-way ANOVA with Dunnett´s post hoc test, using P6 BAT as reference group, # *p* < 0.05, ## *p* < 0.01, ### *p* < 0.001, Student’s unpaired, two-tailed *t*-test.

We then focused on the expression of the so-called brown and beige marker genes. These genes have been identified by transcriptional profiling of the adipose tissue in mice challenged with cold. The most relevant thermogenic gene of mammals is *Ucp1*, and its expression was abundant in both P6 and P56 BAT. *Ucp1* was minimally expressed in iWAT, relative to its levels measured in BAT, but it was a prevalent transcript in P6 iWAT (Figure 5a). The key mitobiogenesis gene *Ppargc1a* was highly expressed in P6 and P56 BAT, and had a moderate and similar level in P6 and P56 iWAT. Other genes associated with adipose tissue “browning”, such as *Cidea*, *Cox7a1* and *Dio2* [72], were abundantly expressed in P56 BAT. Unexpectedly, however, they were much less prevalent in P6 BAT, and were minimally expressed in P56 iWAT. For example, higher levels of *Dio2* were found in P6 iWAT than in P56 iWAT. *Tmem26* and *Tbx1,* which have been previously described as markers of beige adipocytes [40], were abundantly expressed in P6 iWAT (Figure 5a), were less prevalent in P56 iWAT, were minimally expressed in P6 BAT, and were absent in P56 BAT (Figure 5a). These genes were, therefore, unique hallmarks of P6 iWAT. *Eva1a* (encoding Eva-1 homolog A), a transcript previously denoted as a marker of BAT [40,80], was abundant in P6 and P56 BAT, and also in P6 iWAT (Figure 5b), and was significantly less prevalent in P56 iWAT. This gene product is, hence, a shared transcript of BAT and P6 iWAT. 

The majority of brown adipocytes develop from a myogenic factor 5 (Myf5)^+^ cell lineage [47]. We found that the level of *Myf5* was highest in P6 BAT, and was minimally expressed in P56 BAT and P56 iWAT (Figure 5b). Its level was also significantly higher in P6 iWAT than in P56 iWAT (Figure 5b). Another BAT-specific transcript, *Lhx8,* encoding LIM homeobox 8, was most abundant in P6 BAT, less prevalent in P56 BAT, and moderately expressed in P6 and P56 iWAT. There was no difference between *Lhx8* levels in P6 and P56 iWAT (Figure 5b). 

It has been shown that acute cold stress triggers the expression of *Zic1*, encoding zinc finger protein 1, in the subcutaneous fat of mice [40,81]. The level of *Zic1* was prominent in P6 and P56 BAT, and was minimal in P6 and P56 iWAT. Nonetheless, the level of *Zic1* was significantly higher in P6 iWAT than in P56 iWAT (Figure 5b). Two homeobox genes, *Hoxc8* and *Hoxc9,* show increased gene expression during WAT differentiation [40]. We found that both genes were prominently expressed in P56 iWAT, were less expressed in P6 iWAT, and were minimally expressed or lacking in BAT (Figure 5b). 

We also tested for two other potential markers of thermogenic fat development: *Prdm16* and *Cited1*, encoding PR/SET domain 16 and the transcriptional coactivator Cbp/p300-interacting transactivator with Glu/Asp-rich carboxy-terminal domain 1, respectively. PRDM16 promotes BAT differentiation and supports thermogenic adipocyte development [82,83] and “browning” [84], and also maintains BAT in adult mice [85]. We found that *Prdm16* was mostly expressed by P56 BAT, and was expressed to a greater level in P6 iWAT than in P56 iWAT (Figure S2). *Cited1* was originally identified as a beige adipocyte marker [41]; however, whether beige and white adipocytes differ in their *Cited1* expression levels has recently been challenged [33,55]. P6 BAT and P6 iWAT had the strongest *Cited1* expression, and P56 BAT had a low *Cited1* level (Figure S2). 

We recently showed that ether lipids, the so-called alkylglycerols (AKGs), sustain beige adipogenesis in the subcutaneous adipose tissue of newborn mice, and that AKG monooxygenase (AGMO) inhibits their function by metabolizing them [38]. We found that the level of *Agmo* was high in P56 BAT and P56 iWAT, and was moderate in P6 BAT and P6 iWAT (Figure 5b). 

We next analyzed the transcription of cell membrane receptor genes that are required for brown and beige adipogenesis. Cold stress induces beige adipogenesis through β-adrenergic receptors (Figure 5c). We found that these receptors were expressed mostly by P56 BAT and P56 iWAT (Figure 5d). Indeed, *Adrb3*, encoding ADRB3, an adrenergic receptor involved in cold-induced adipose tissue browning, was highly expressed in P56 iWAT, and was moderately detectable in P6 iWAT and in P56 BAT (Figure 5e). The mouse 3T3-L1 preadipocyte line lacked *Adrb3* expression, and this remained unchanged during adipocyte differentiation (Figure 5f), which accords with the lack of beige induction in 3T3-L1 cells by certain stimuli [86]. Unexpectedly, however, primary mouse preadipocytes also lacked *Adrb3* expression, although its expression was strongly increased during adipocyte differentiation (Figure 5f). Genes of the β-adrenergic signaling pathway were not expressed in P6 iWAT or in nondifferentiated preadipocytes, suggesting that beige induction by noradrenaline is a trait of adult adipose tissue. 

It is known that the Th2 cytokines IL-4 and IL-13 induce beige adipocyte differentiation, and that IL-4 receptor alpha (IL4Rα) is necessary for cold-induced beige adipocyte development [87]. It is thought that the Th2 cytokines are released into the adipose tissue stroma by mast cells or eosinophils in response to cold (Figure 5g) [87,88]. However, mast cells were scarce in BAT (Figure 5h), and a similar paucity of eosinophils in BAT has been reported previously [58]. Moreover, *Il4ra* was moderately expressed by P6 BAT, P56 BAT and P6 iWAT, whereas P56 iWAT showed abundant *Il4ra* expression (Figure 5i). The expression level of *Il4i1,* encoding interleukin 4-induced 1 protein, had a similar, even stronger pattern: P56 iWAT showed abundant *Il4i1* expression, whereas P6 BAT, P56 BAT and P6 iWAT had negligible *Il4i1* levels (Figure 5i). The expression level of *Rnf128*, which is responsible for the inactivation of the downstream IL4Rα signaling [57], had an inverse pattern, with the highest level in P6 BAT and the lowest level in P56 iWAT (Figure 5i). These data reveal that the IL-4/IL4Rα/STAT6 signaling is repressed in BAT and P6 iWAT.

The above two signaling mechanisms have been established in adult mice, and are known as inducers of beige adipogenesis under cold stress. Much less is known about the signals that induce thermogenic fat in newborns. We have shown recently that signaling through the platelet-activating factor (PAF) receptor (PTAFR) maintains beige adipogenesis in newborn mice (Figure 5j) [38]. In line with this, *Ptafr* expression was highest in P6 BAT and P6 iWAT, with minimal or moderate expression in P56 BAT and P56 iWAT, respectively (Figure 5k,l).

In summary, our findings reveal that receptors for beige adipogenesis are distinctly expressed in adult and in infant adipose tissue. β-adrenergic signaling is predominant in P56 iWAT. Functional IL-4 signaling is evident in P56 iWAT, but not in P6 iWAT (Figure 5l). Lastly, the necessary genes for the recently described AKG-, and PAF-mediated beige adipogenesis program are highly expressed in P6 iWAT (Figure 5b,k,l).

## 4. Discussion

In the present study we have identified gene networks and hub genes that are hallmarks of the developing BAT and iWAT. We found that the transcriptional landscapes of young and adult BAT are markedly different, with an overrepresentation of thermogenic and lipid catabolism transcripts in adult BAT. This is an unexpected finding, as thermogenic BAT is morphologically mature at birth (Figure 1a) [72], and it was thought to be relevant for thermogenesis of the infant [31]. We also found that young iWAT expresses thermogenic and lipid catabolism transcripts together with an abundance of transcripts associated with cold-induced beige adipogenesis. This indicates that BAT is still undergoing differentiation and maturation in newborn mice, and that iWAT temporally adopts thermogenic potential. In line with this, we found that iWAT of young mice has a beige/brite morphology and a gene expression landscape of beige/brite fat. However, the beige-inducing signal pathways are distinct in adult and young iWAT, highlighting that the signals for thermogenic fat differentiation may be different in early postnatal life and in adulthood. 

Adipose tissue development in the first three years of life is known to be key for determining childhood obesity [89]. It is also known that obese children lose their thermogenic fat cells early on, whereas lean children maintain them in their subcutaneous fat depots until puberty [31,56]. Breast milk-derived signals play a key role in triggering and maintaining beige adipogenesis in infant fat [38]. Moreover, the subcutaneous adipose tissue of the infant has lipolytic activity and releases free fatty acids, but this process declines in childhood obesity [52]. It is thus key to maintain the infant-type features of the adipose tissue, including its beige adipocyte-like traits in early postnatal life to prevent childhood obesity.

We have identified novel hub genes and gene networks that can be used to discriminate between both young and adult BAT and iWAT. Because obesity reduces BAT mass, and a premature loss of beige adipocytes may contribute to obesity [38,56], adequate animal models and experimental approaches are needed to study the mechanisms which sustain BAT and beige adipocytes. The novel hub genes and gene networks not only decipher as yet unexplored functions of the BAT and beige adipocytes, but will also aid future studies by providing target genes and gene networks to analyze by transcriptional profiling or single-cell sequencing. 

Hallmark genes and gene networks of the young iWAT were associated with cell−cell adhesion and cell−matrix interaction, with the cytoskeletal protein *Krt5* acting as a hub gene. KRT5 is a protein of the basal layer of the skin epithelia. While there is close proximity between iWAT and the skin, it is unlikely that keratinocytes are connected to the developing fat. However, some fat cell lineages, including BAT adipocytes, are derived from the embryonic ectoderm [90], which is rich in keratin expression [91]. Moreover, *Krt5* is expressed by myoepithelial progenitors [92], and BAT fat cells have been shown to develop from myoepithelial cells in the mammary gland [93] where epithelium-to-adipocyte transdifferentiation might occur [94,95]. It is known that *Krt19* is expressed by mouse preadipocytes, and in vitro adipocyte differentiation is associated with an increment in *Krt19* expression [46]. *Krt19* was overrepresented in P6 iWAT and belonged to the *Krt5* gene network. Moreover, the levels of *Krt5* and several keratin and desmosome genes are suppressed in obesity and in type 1 diabetes in the mouse, leading to obesity-associated skin fragility [96,97]. Keratin also increases the adipogenic differentiation of adipose tissue stem cells [98]. Our findings suggest that early fat development may be associated with keratins through as yet unknown mechanisms.

Steroidogenesis genes were underrepresented in P6 iWAT. Adipocytes are steroidogenic cells, and are capable of de novo production of pregnenolone and the oxysterol 27-hydroxycholesterol from cholesterol and its precursor, mevalonate. The inhibition of steroidogenesis induces adipocyte differentiation [99]. Adipocyte differentiation in human subcutaneous fat is associated with an increase in the mRNA levels of enzymes synthesizing and inactivating androgens [100], and iWAT acquires its steroidogenic potential during its maturation. As young iWAT shows features of beige adipocytes, the marked difference in the steroidogenic potential of young and adult iWAT has relevance for childhood obesity. The premature loss of beige adipocytes has been observed in obese children, and as a result of insufficient breastfeeding [38,56]. Steroidogenesis, in particular the level of sexual steroids, is key in the early life imprinting of the sex-dependent differentiation of organs. In the first six months of life in boys, and during the first two years in girls, there is a transient sex-specific activation of the hypothalamic−pituitary−gonadal axis, which is called minipuberty [101]. Minipuberty is associated with the rise in estradiol and testosterone, and is thought to determine sex-specific growth and maturation in later life. Moreover, minipuberty influences body composition [101]. As adiposity in infancy has an impact on obesity status in adulthood, the premature loss of beige adipocytes may compromise minipuberty and trigger obesogenic imprinting during metabolic development. Insufficient breastfeeding impairs several physiological functions that are associated with minipuberty, such as body adiposity, cognitive development and gonadal development [38,102,103]. It is plausible that breastfeeding is required for postnatal adipose tissue functions, which contribute to the hormonal changes of the minipuberty.

Young BAT and young iWAT shared a similar expression of a local angiotensin-kininogen system, inhibitory G-protein-linked neuropeptide receptors and GABA-A signaling. Specifically, NPY receptors, *Npy*, *Gal*, *Npbwr1* and various components of the GABA-A receptor signaling were overrepresented DEGs in young BAT and iWAT. GABA-A receptors were also overrepresented in adult BAT. NPY2R is the receptor for NPY, which is an important central orexigenic hormone that is also produced by the subcutaneous fat [104], and cold stress induces NPY release [105]. It is therefore important that young iWAT expresses higher levels of *Npy* and *Npy2r* than adult iWAT or BAT, suggesting that fat-derived NPY may have a role in the postnatal development of the adipose tissue. NPY induces angiogenesis and fat cell differentiation through NPY2R [105], making it plausible that NPY may be responsible for early vascularization and expansion of iWAT. In adults, however, excessive NPY signaling leads to obesity [106,107] and NPY increases adipocyte size in hyperinsulinemic conditions [104]. Young iWAT also showed elevated expression of *Npy4r*, which encodes NPY4R, a receptor for PP. PP is released from the pancreas in response to meal ingestion and reduces appetite. Low plasma PP levels have been observed in human obesity [108,109], and PP levels are higher in men than in women [110]. It is plausible that PP, similarly to NPY, has a role in early fat development. The transcript of the *Npy6r* pseudogene was also enriched in young iWAT, however, its gene product has no ligand binding for NPY or PP [111]. Young iWAT also showed increased expression of the neuropeptide receptors NPBWR and NPFFR1. NPBWR is a receptor for the neuropeptide W and neuropeptide B. Ablation of this receptor in mice leads to obesity [112], and NPBWR signaling has a role in the central regulation of energy balance [113]. Similarly, NPFF in the hypothalamus suppresses appetite [114], and protects from obesity-induced inflammation, and obesity is associated with decreased plasma NPFF levels [57,115].

Interactome maps of the neuropeptide signaling gene networks of young iWAT and BAT pointed to their association with NFκB and STAT3 signaling. Suppressed chemotaxis for granulocytes and underrepresented NFκB signaling were shared features of young BAT and iWAT. This immune-suppressed state was retained in adult BAT, which showed very little immune cell content. By contrast, maturation of iWAT was associated with the overrepresentation of inflammatory pathways and the decline in the expression of the local angiotensin-kininogen system, inhibitory G-protein linked neuropeptide receptors and GABA-A signaling. GABA has a role in suppressing inflammatory cytokine signaling [116,117,118]; however, its effects are dependent on the GABA receptor engaged. For instance, the GABA-B receptor, which showed a stable expression in young and adult adipose tissue, worsens BAT function, and inhibition of this signaling axis restores BAT in obese mice [119]. Unexpectedly, young iWAT had a strongly expressed innate immune gene network, involving cathelicidin and β-defensin [120]. Young iWAT was also unique in the expression of PIWI genes, which are involved in the neutralization of transposable elements, safeguard genome integrity and ensure the adaptation of the immune response to rapidly evolving viruses [121]. 

We also identified some unexplored traits of adult BAT, which uniquely expressed vitamin K-dependent protein C and coagulation factor XII (Hageman factor). Vitamin K-dependent protein C is an anticoagulation factor, and is activated by the thrombin-thrombomodulin complex to degrade the activated forms of coagulation factors V and VIII. Mutations in the gene are associated with thrombophilia, neonatal purpura fulminans, and recurrent venous thrombosis [122,123]. Hageman factor activates the intrinsic pathway of the blood coagulation cascade, and is also involved in fibrinolysis. Of relevance, obesity is associated with metainflammation and hyperlipidemia, which both contribute to thrombotic disorders and the dysregulation of endogenous anticoagulant mechanisms [124]. For instance, unsaturated fatty acids and long-chain fatty acids are known to activate the Hageman factor [125,126], while metainflammation is associated with an increased risk of thrombosis [127]. BAT may have an as yet unexplored role in hemostasis, and this may be impaired by the loss of BAT in obesity. 

## 5. Conclusions

**Figure 6:**
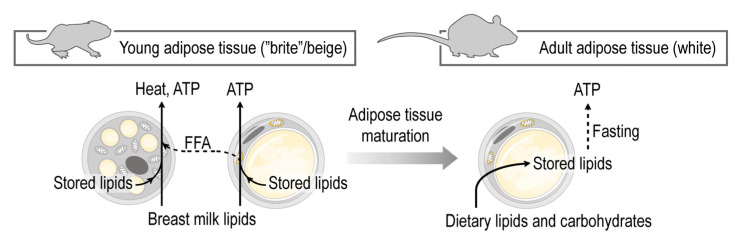
Maturation of the subcutaneous adipose tissue entails a shift from fat catabolism and thermogenesis to fat storage. The subcutaneous adipose tissue in early postnatal life shows hallmarks of beige adipocytes, and supports the catabolism of dietary lipids, mostly breast milk lipids. At this developmental stage the fat is catabolized by the adipocytes to elaborate free fatty acids (FFA), produce energy and heat. Breast milk lipid mediators maintain beige adipocytes. In adulthood, however, the subcutaneous adipose tissue is predominantly a lipid storage site.

We found that the iWAT of young mice is rich in beige fat cells and can be defined as a “bona fide” beige adipocyte depot (Figure 6). Metabolic genes including thermogenic and lipid catabolism genes were expressed in young iWAT, which likely means that this fat depot functions to break down fat to produce energy and heat. Lipolysis is a trait of beige adipocytes and BAT, and the inhibition of lipolysis favors thermogenic fat development [37], with lipid metabolites functioning as key signals for beige adipocyte, and BAT differentiation [36]. Breast milk-derived lipid mediators are known to sustain thermogenic fat in the infant [38,128]. By contrast, the transcriptional landscape of adult iWAT is associated with lipid storage, and the inactivation of beige adipocyte-inducing lipid species [38].

Our findings indicate the probable existence of metabolic reprogramming during the maturation of subcutaneous adipose tissue, shifting from the burning of fat as heat and energy to the accumulation of lipids as energy storage. This metabolic shift is in good agreement with the previously observed intensive lipid catabolism and fatty acid release from the subcutaneous adipose tissue in newborn mammals, including human infants [14,19,25,54], and the potential of the subcutaneous adipose tissue-derived adipocytes to transform into beige adipocytes in response to cold stress [44,72]. 

Indeed, the expression of beige adipocyte genes and the multilocular appearance of fat cells in the subcutaneous adipose tissue of human infants support the presence of subcutaneous beige adipocytes in humans [38,56]. The existence of a subcutaneous beige adipocytes is relevant in the early life determination of obesity, as signals triggering the premature loss of beige adipocytes may be the roots of a premature expansion of the WAT, leading to childhood obesity. 

## Data Availability

Raw NGS data are deposited at GEO with the accession number #GSE133500. For secondary analysis, we used our previously published NGS dataset, with accession number #GSE125405. Flow Repository identifier of raw FACS data: #FR-FCM-Z236.

## Supplementary Materials

The following are available online at https://www.mdpi.com/article/10.3390/cells10092368/s1, Figure S1: Workflow of our NGS analysis and correlation heatmap of the samples analyzed, Figure S2: Area of beige fat in the iWAT at P6 and P56, Figure S3: Interactive maps of gene networks, Figure S4: Immunostaining of GABA_B_R in P6 and P56 iWAT, Table S1: *Krt5* network, Table S2: *Mup2* network, Table S3: *Agtr2* network, Table S4: *H2-Q9* network, Table S5: *Ucp1* network, Table S6: *Krt71* network, Table S7: *Fabp3* network, Table S8: *Glycam1* network.
