# Supplementary material for: Transcriptional Landscaping Identifies a Beige Adipocyte Depot in the Newborn Mouse
Source: Cells. 2021 Sep 9;10(9):2368. doi: 10.3390/cells10092368 (PMC8470180; doi:10.3390/cells10092368)
Supplement: Supplementary file 1 [file cells-10-02368-s001.zip › cells-1354767 supplementary 3/cells-1354767 supplementary figures.pdf]

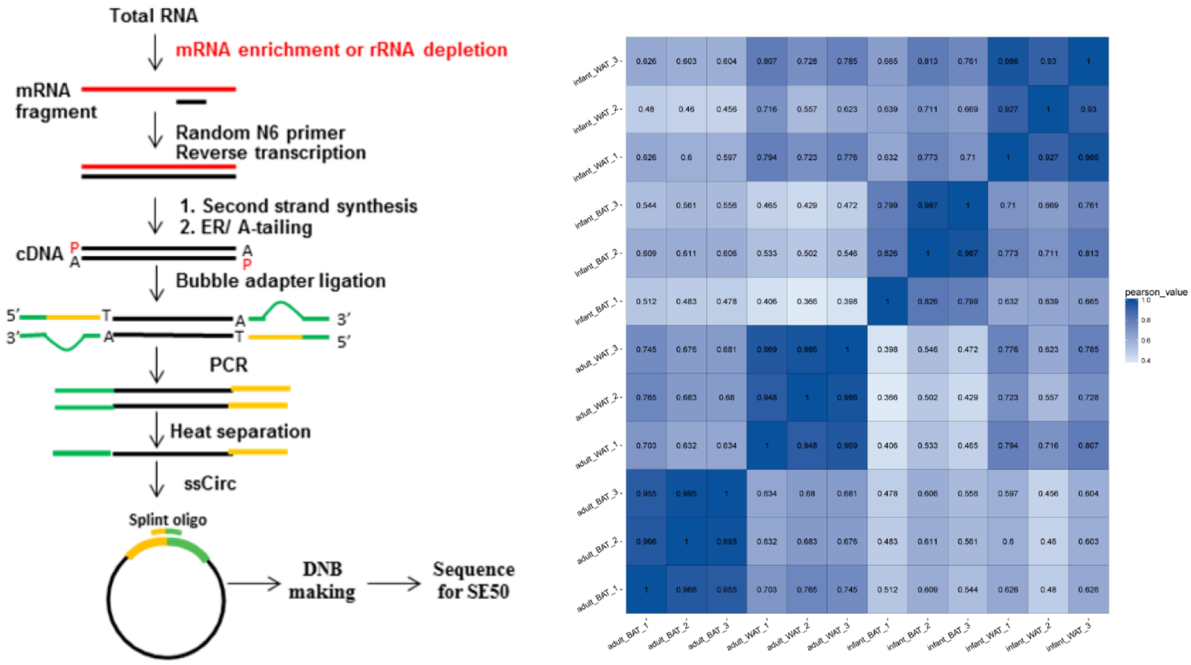

Figure S1. Workflow of the NGS analysis *(Left)*,
and correlation heatmap of the samples analyzed *(Right)*

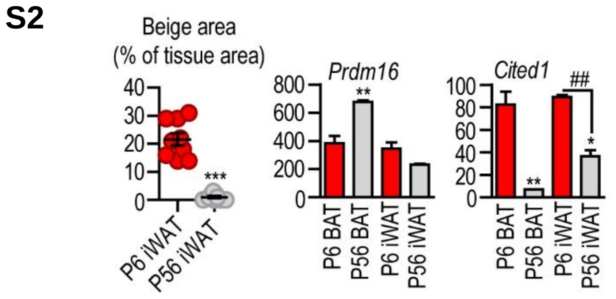

Figure S2. Area of beige adipocyte-rich regions in the iWAT at P6 and P56

Beige adipocyte-rich area was calculated with BeAR© image analysis, using H&E stained sections, as described (Yu et al 2019) . ***p<0.001, Student's unpaired, two-tailed t-test Relative expression of *Prdm16* and *Cited1*. **p<0.01, *p<0.05, one-way ANOVA with Dunnett's post hoc test; ## p<0.01 Student's unpaired, two-tailed t-test

Figure S3. Interactive maps of gene networks

Step 1: Download the raw files, or open the interactive gene network files, using this link.

Step 2. Save the files in the following order of library structure:

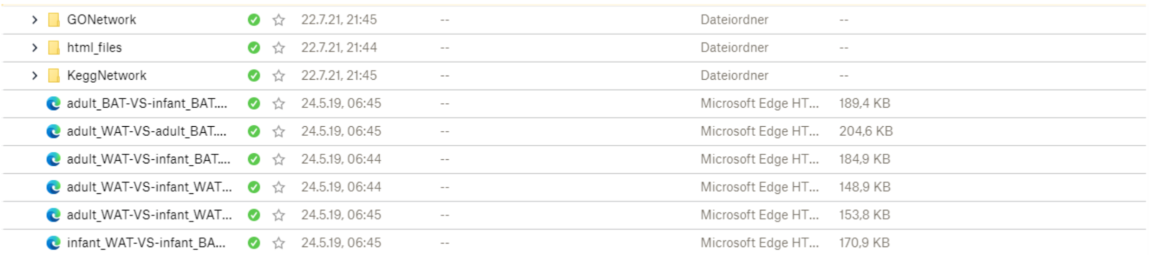

Step 3. Open the interactive map, select the desired GO or cellular process, and the gene or gene network of interest. Examples of network analyses are shown below.

Example 1

Metabolic pathways and their interconnections, with individual gene names

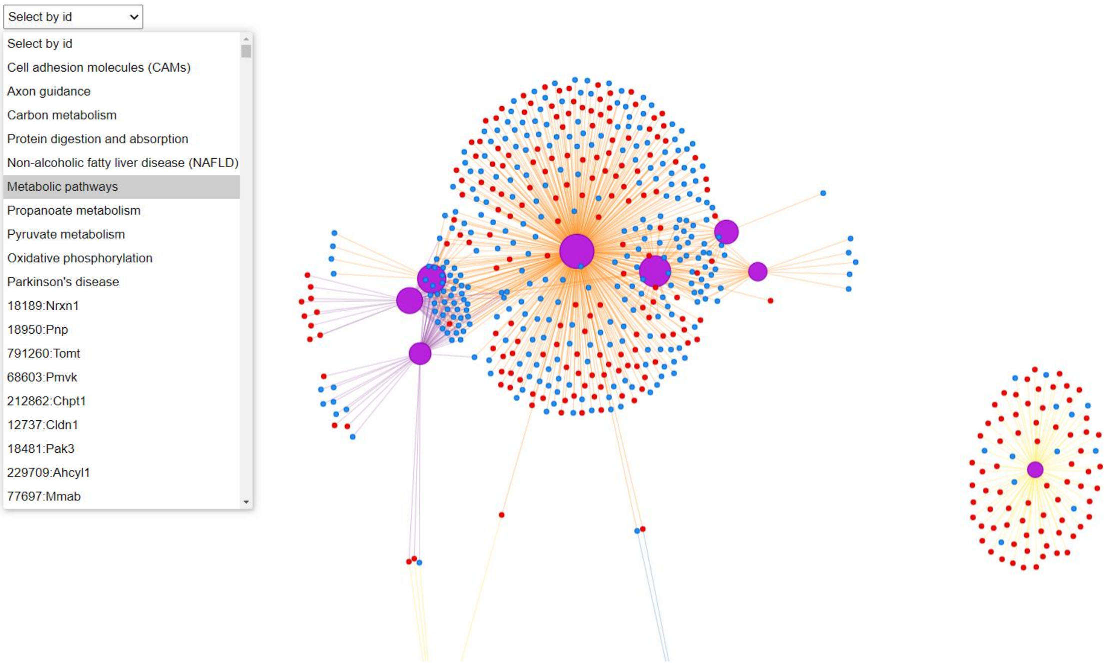

Example 2

KEGG pathway networks

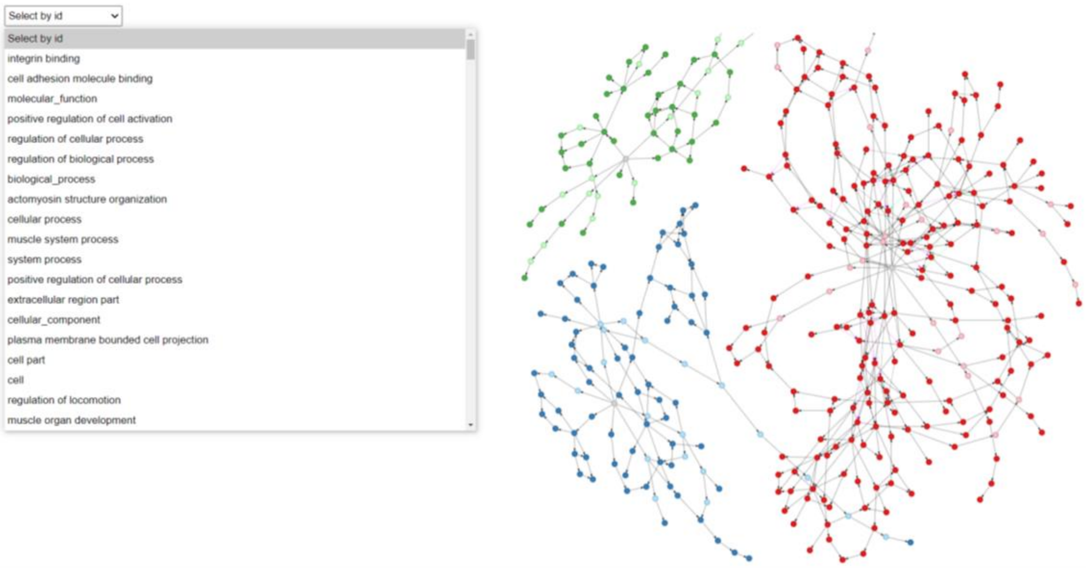

S4

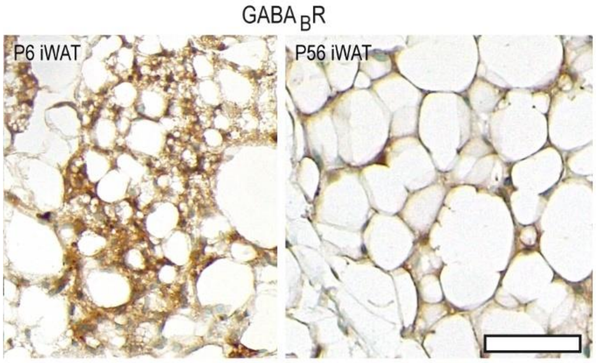

Figure S4. Immunostaining of $GABA_BR$ in P6 and P56 iWAT, scale 50 μm.

Reference

Yu H, Dilbaz S, Coßmann J, Hoang AC, Diedrich V, et al. 2019. Breast milk alkylglycerols sustain beige adipocytes through adipose tissue macrophages. *The Journal of Clinical Investigation* 129: 2485-99
